# Pre-Exposure Prophylaxis YouTube Videos: Content Evaluation

**DOI:** 10.2196/publichealth.7733

**Published:** 2018-02-16

**Authors:** Aleksandar Kecojevic, Corey Basch, Charles Basch, William Kernan

**Affiliations:** ^1^ Department of Public Health William Paterson University Wayne, NJ United States; ^2^ Teachers College Columbia University New York City, NY United States

**Keywords:** YouTube, pre-exposure prophylaxis (PrEP), Truvada, video content.

## Abstract

**Background:**

Antiretroviral (ARV) medicines reduce the risk of transmitting the HIV virus and are recommended as daily pre-exposure prophylaxis (PrEP) in combination with safer sex practices for HIV-negative individuals at a high risk for infection, but are underused in HIV prevention. Previous literature suggests that YouTube is extensively used to share health information. While pre-exposure prophylaxis (PrEP) is a novel and promising approach to HIV prevention, there is limited understanding of YouTube videos as a source of information on PrEP.

**Objective:**

The objective of this study was to describe the sources, characteristics, and content of the most widely viewed PrEP YouTube videos published up to October 1, 2016.

**Methods:**

The keywords “pre-exposure prophylaxis” and “Truvada” were used to find 217 videos with a view count >100. Videos were coded for source, view count, length, number of comments, and selected aspects of content. Videos were also assessed for the most likely target audience.

**Results:**

The total cumulative number of views was >2.3 million, however, a single Centers for Disease Control and Prevention video accounted for >1.2 million of the total cumulative views. A great majority (181/217, 83.4%) of the videos promoted the use of PrEP, whereas 60.8% (132/217) identified the specific target audience. In contrast, only 35.9% (78/217) of the videos mentioned how to obtain PrEP, whereas less than one third addressed the costs, side effects, and safety aspects relating to PrEP. Medical and academic institutions were the sources of the largest number of videos (66/217, 30.4%), followed by consumers (63/217, 29.0%), community-based organizations (CBO; 48/217, 22.1%), and media (40/217, 18.4%). Videos uploaded by the media sources were more likely to discuss the cost of PrEP (*P*<.001), whereas the use of PrEP was less likely to be promoted in videos uploaded by individual consumers (*P*=.002) and more likely to be promoted in videos originated by CBOs (*P*=.009). The most common target audience for the videos was gay and bisexual men.

**Conclusions:**

YouTube videos can be used to share reliable PrEP information with individuals. Further research is needed to identify the best practices for using this medium to promote and increase PrEP uptake.

## Introduction

Between 2005 and 2015, the number of people diagnosed with HIV in the United States has declined substantially (19%) [1]. Prevention efforts have reduced HIV infection rates in several key populations, including people who inject drugs (PWID), heterosexuals, and African Americans. However, HIV remains a persistent problem among some population subgroups, particularly among men who have sex with other men (MSM), where the number of new infections has increased by 9% between 2010 and 2014 [2]. Worldwide, an estimated 35 million people live with HIV, the majority of whom live in Sub-Saharan Africa [3].

Recent scientific advancements have provided additional prevention options with the potential to reduce rates of new infections. Early treatment with antiretroviral (ARV) medicines improves the health of people living with HIV and reduces the risk of transmitting the virus by 96% [4]. Furthermore, findings from several clinical trials [5-7] led the Food and Drug Administration (FDA) to approve use of once daily ARV (Truvada) as the pre-exposure prophylaxis (PrEP) to be used in combination with safer sex practices for HIV-negative individuals at a high risk for infection [8]. The World Health Organization (WHO) recommended PrEP as an additional prevention strategy for any person at a substantial risk of contracting HIV [9]. While PrEP can be a viable approach to controlling the spread of HIV, it is not being used to its full potential. Certain estimates suggest that too few people in the United States are taking PrEP, whereas many others at a risk of contracting HIV are not even aware of PrEP [10]. Although, awareness of PrEP has increased among highly sexually active MSM [11,12], uptake has been slow, particularly among younger MSM and MSM of color [13,14]. Researchers have hypothesized that the slow uptake of PrEP may be due to cost [15], lack of awareness coupled with the belief that PrEP is for only for those who engage in high-risk behaviors [16], stigma [17], psychological and social barriers [18], or even provider-initiated barriers [19]. Worldwide, most countries have not taken any steps yet, due to concerns about local relevance, costs and sustainable funding, and other health system issues [20,21]. Some researchers state that communicating reliable information about PrEP to the public is not straightforward, as people may not be willing to seek information about PrEP through their close interpersonal networks [22] or even through their health care providers [23]. Researchers have argued that the effectiveness of PrEP will depend largely on the informed involvement of various stakeholders, including government officials, primary care providers, recipients, and community educators [22,24].

Previous communication research has shown that mediated interaction can reflect and shape popular understanding of important health issues [25,26]. The internet, including social media, is now used extensively not only to communicate but also to search for health information [27,28]. Video-sharing sites such as YouTube are among the most popular websites with over a billion users and hundreds of millions of hours of content [29]. YouTube also contains a vast amount of videos pertaining to health information, including information about HIV and HIV prevention. For many, particularly the younger audiences and sexual and racial minorities, YouTube can serve as a platform where users seek PrEP information, generate content, share information within their networks, and disseminate content to reach a wider audience [30,31]. An additional feature of the video-sharing format is its capacity for timely updates. A nationwide survey focusing on how adults in the United States use Web-based resources for health information found that 26% watched someone else’s experience about a health issue [32]. In other parts of the globe, internet users are also seeking health information using social media [33].

Health professionals are becoming increasingly aware of the fact that health consumers use social media to gather health information. Consequently, examining social media to understand the content of health information has become a growing area of public health research. For example, video content on YouTube has been analyzed on a variety of topics, including the viral pandemics [34,35], contraception [36], electronic cigarettes [37], cancer [38,39], rheumatoid arthritis [40], or immunizations [41-44]. While social media could represent critical communication channels for increasing awareness and interest about health issues, previous research shows that health information posted on such media can be incorrect or misleading [45,46]. Videos uploaded by the lay public not only contained information that contradicts public health guidelines but in some cases received high view counts and user ratings [47,48]. Hence, studying health content on social media is important to understand these novel forms of health information dissemination [49].

To our knowledge, no published study investigated PrEP-related information that is disseminated through YouTube videos. The primary purpose of this study was to describe the sources, characteristics, and content of the most widely viewed YouTube videos associated with PrEP. Understanding what information on PrEP is currently available on social media such as YouTube, whether users are seeking information on PrEP through this source, and who is generating this information may help HIV prevention efforts to tailor messages, promote more effective knowledge translation, and increase the rates of PrEP uptake.

## Methods

### Search Strategy

A search of YouTube was conducted using two terms: “pre-exposure prophylaxis” and “Truvada.” The YouTube interface provides an approximation of the number of videos retrieved for a keyword search (eg, typing “pre-exposure prophylaxis” into the YouTube search bar yielded approximately 5200 results on September 23, 2016). We were interested in the most viewed videos (as determined by filtering and sorting by the number of views on YouTube), hence search results were limited to videos that were viewed 100 times or more. A single YouTube account’s viewing history was used to document the videos examined. Videos were excluded if they were not in English. Country of origin was determined by the source information provided in the YouTube video description. Duplicate videos were excluded, as were those with no accompanying audio. The final tally included 217 unique videos retrieved by one or both keywords (ie, “pre-exposure prophylaxis” and “Truvada”). Unique videos were then reviewed to assess their relevance to PrEP. This study was not classified as being a human subject’s research by the institutional review boards at William Paterson University and Teachers College because it involved use of public access data.

### Data Review and Analysis

Video data were coded into an Excel (Microsoft Corporation, Redmond, Washington) spreadsheet and analyzed by a trained research assistant (RA). We recorded the objective characteristics of each video, including video title, URL, date of upload, length of the video, number of views, number of likes and dislikes, number of comments posted by YouTube users, and descriptive text included by the user who uploaded the video. Sources of information were defined as the following: (1) individual consumer (information provided by an individual with no professional credentials or established organizational affiliation), (2) institutional (information provided by individuals with professional credentials, eg, medical doctor [MD], registered nurse [RN], established organizational affiliations such as academic organization, or any government organization, eg, Centers for Disease Control and Prevention [CDC]), (3) news or media (information provided by network or internet-based news organization, eg, American Broadcasting Company [ABC]), and (4) community-based organizations (CBO; information provided by CBO or their representatives).

Seven content categories were identified a priori using the CDC fact sheet on PrEP [10], literature related to PrEP and social media [22], and previous studies exploring the content of YouTube videos [50-52]. These categories included (see Table 1): (1) defining PrEP (ie, prevention tool used to reduce the risk of HIV infection); (2) explaining how PrEP works; (3) describing who can use PrEP; (4) mentioning PrEP as a safe treatment; (5) describing PrEP side effects; (6) describing how to obtain PrEP, and (7) mentioning PrEP costs. Finally, we assessed whether the video promoted use of PrEP as a prevention tool. All content categories were coded dichotomously (0=no, 1=yes).

Additionally, the study team was interested in the visual representation of the content. On the basis of who the presenter of the PrEP-related information was, all videos were coded into 5 categories: (1) a single individual, (2) multiple individuals, (3) animations/videos/advertisements, (4) newsreels, and (5) other (ie, scientific slides presentation). On the basis of the overall narrative, visuals, description, and the source of each video, the most likely target audience for each video was documented (ie, MSM, racial minority MSM, transgender population, women and heterosexual couples regardless of race/ethnicity, scientific/professional audience, nonspecific/anyone who may benefit/anyone at risk of contracting HIV). For example, if gay African American men narrated their PrEP experience, these videos were classified as more likely to target racial minority MSM. Similarly, if the source of the video was the Ball community, it was labeled as more likely to appeal to racial minority MSM. The study team was particularly interested in videos aimed at racial minority MSM due to the known slower uptake of PrEP by men of color. If the video discussed more than one of the above groups as a beneficiary of PrEP (ie, gay men, sex workers, transgender population, PWID, heterosexual couples in serodiscordant relationship), the video was labeled as targeting anyone who may benefit from PrEP or anyone at risk of contracting HIV.”

To ensure consistency in coding, the first 10 videos were coded collaboratively by the first author and the RA until a consensus was reached. All analyses of the video and content characteristics were performed using SPSS 23.0 (IBM Corporation). Given that the video lengths, the number of views and comments were not normally distributed, analyses used the Kruskal-Wallis test, followed by post hoc tests, to examine the differences in video characteristics between different sources. To examine differences in content categories among different sources, we calculated frequencies and percentages, medians and ranges, and chi-square tests of independence followed by post hoc tests using adjusted standardized residuals with Bonferroni corrections of the *P* values [53]. An alpha level of .05 was used to determine the significance for all tests.

**Table 1:** Pre-exposure prophylaxis (PrEP)-related content categories, definitions, and examples.

Category	Definition	Example
Defines/explains PrEP	The taking of a prescription drug as a means of preventing HIV infection in an HIV-negative person.	“An option for someone who is HIV negative, but who is at a substantial risk of contracting it, to prevent HIV infection.”
Describes how PrEP works	By taking Truvada (a combination of 2 drugs, tenofovir and emtricitabine) daily, the presence of the medicine in the bloodstream can stop HIV from taking hold and spreading in the body.	“HIV-negative individuals use Truvada daily to reduce their risk of becoming infected. It works to prevent HIV from establishing infection inside the body.”
Describes who can use PrEP	Claiming or mentioning who should receive PrEP	“Truvada prevents infection in sexually active adults.”
Promoted PrEP as a safe, effective option	Discussing the effectiveness of using PrEP to prevent HIV	“You take one pill a day, and you stay HIV-negative.”
Discusses side effects	Mentioning side effects from taking PrEP/Truvada	“There are concerns about increased kidney function and decreased bone mineral density.”
Describes how to obtain PrEP	Mentioning where and/or how to obtain PrEP	“PrEP can be prescribed only by a doctor, so talk to yours to find out if PrEP is the right thing for you”
Discusses the cost of PrEP	Mentioning cost of PrEP and if insurance covers it	“My insurance covers Truvada, I paid copay only.”
Promotes use of PrEP	Encouraging PrEP use among those at risk	“People at a risk should take a pill every day.”

## Results

**Figure 1:**
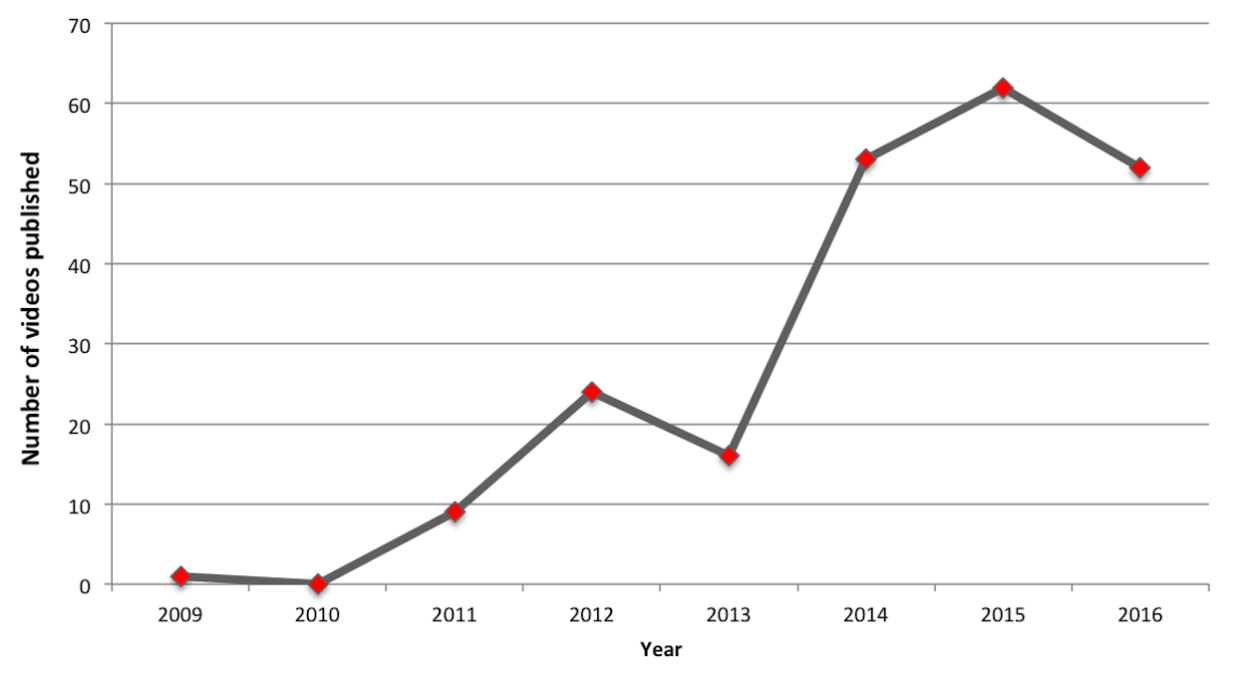
Number of pre-exposure prophylaxis (PrEP) YouTube videos published per year (until October 1, 2016).

Figure 1 presents the number of PrEP YouTube videos published per year. We observed a significant increase in the number of published videos from 2013 to 2014. The 217 PrEP videos identified in this study were posted from 189 unique YouTube accounts. The overwhelming majority of videos originated in the United States (171/217, 78.8%), followed by Canada (18/217, 8.2%), the United Kingdom, and Australia (each 8/217, 3.7%). The remainder of the videos (12/217, 5.4%) originated in the rest of the world, including a few international organizations.

Collectively, these videos were viewed 2,369,003 times, however, a single CDC video accounted for over 1.2 million views. This video was animation accompanied by voiceover, published on January 7, 2016 and was 2 minutes and 51 seconds in length. The second most viewed video was viewed over 193,000 times and originated by the online media source, VICE. This video was published on June 26, 2015 and was 27 minutes and 10 seconds in length.

Characteristics of videos classified according to their source are described in Table 2. Institutions (66/217, 30.4%) and consumers (63/217, 29.0%) presented the largest number of videos followed by CBOs (48/217, 22.1%) and media (40/217, 18.4%). While no significant differences in the length of videos between different sources were observed, the Kruskal-Wallis test indicated significant differences in both number of views (*P*=.003) and number of comments (*P*<.001) between different sources. With regard to the number of views, the institution videos had a mean rank significantly lower than the consumer (*P*=.05) and media (*P*=.02) videos, adjusted for multiple comparisons. With regard to the number of comments, the consumer videos had a mean rank significantly higher than the institution (*P*<.001) and the CBO (*P*<.001) videos, while media videos had a mean rank significantly higher than the institution (*P*=.002) videos, all adjusted for multiple comparisons.

**Table 2:** Characteristics (length, number of views, and number of comments) of the most viewed pre-exposure prophylaxis (PrEP) YouTube videos by their sources.

Video characteristics	Consumer (N=63)	Institutions^a^ (N=66)	Media (N=40)	Community-based organization (N=48)	Total (N=217)	Kruskal-Wallis test—H (degrees of freedom); *P* value
Length in mm:ss, median (IQR^b^)	05:35, (02:13-09:57)	05:21, (01:55-16:55)	04:01 (02:30-06:14)	04:06, (01:38-09:57)	04:40, (02:15-09:57)	2.2 (3); .53
Number of views, median (IQR)	728^c^, (371-4097)	412^d^, (210-774)	881^c^, (281-9449)	353^c,d^, (158-1419)	520, (253-2416)	13.9 (3); .003
Number of comments, median (IQR)	4^e^ (1-23)	0^f^ (0-1)	1^e,g^ (0-12)	0^f,g^ (0-1)	1 (0-4)	43.6 (3); <.001

^a^Government, health or academic professional.

^b^IQR: interquartile range.

^c,d^Superscript letters indicate classes of information providers whose mean ranks for views do not differ significantly from each other at alpha=.05, following post hoc tests.

^e,f,g^Superscript letters indicate classes of information providers whose mean ranks for comments do not differ significantly from each other at alpha=.05, following post hoc tests.

**Table 3:** Content characteristics of the most viewed YouTube pre-exposure prophylaxis (PrEP) videos by their sources.

Content categories	Consumer (N=63), n (%)	Institutions^a^ (N=66), n (%)	Media (N=40), n (%)	Community-based organization (N=48), n (%)	Total (N=217), n (%)	Chi-square test (degrees of freedom); *P* value
Defines PrEP	47 (74)	55 (83)	38 (95)	40 (83)	180 (82.9)	7.2 (3); .07
Describes how PrEP works	27 (42)	37 (56)	18 (45)	25 (52)	107 (49.3)	2.7 (3); .44
Describes who can use PrEP	34 (54)	41 (62)	31 (77)	26 (54)	132 (60.8)	6.9 (3); .08
Promotes PrEP as safe option	11 (17)	16 (24)	11 (27)	12 (25)	50 (23.0)	1.7 (3); .63
Discusses side effects	18 (28)	26 (39)	13 (32)	13 (27)	70 (32.3)	2.5 (3); .48
Describes how to obtain PrEP	22 (34)	22 (33)	13 (32)	21 (43)	78 (35.9)	1.7 (3); .63
Discusses the cost of PrEP	18 (28)^b^	13 (19)^b^	21 (52)	8 (16)^b^	60 (27.6)	17.4 (3); .001
Promotes use of PrEP	45 (71)	58 (87)^c^	32 (80)^c^	46 (95)^c^	181 (83.4)	13.2 (3); .004

^a^Government, health or academic professional.

^b^Indicates information providers whose proportion of videos discussing cost of PrEP do not differ significantly from each other at the alpha=.05, following post hoc tests.

^c^Indicates information providers whose proportion of videos promoting use of PrEP do not differ significantly from each other at the alpha=.05, following post hoc tests.

Overall, more than 80% of the videos defined and promoted the use of PrEP, and more than 60% described who can use PrEP (ie, people who are HIV-negative and want to protect themselves from contracting HIV) (Table 3). In contrast, less than one-third of the videos addressed the cost, side effects, or safety aspects of PrEP. Over one-third of the videos (78/217, 35.9%) discussed how to obtain PrEP, and over one-quarter (60/217, 27.6%) discussed the costs of PrEP. Chi-square analyses were conducted to explore differences among the 4 sources of information in each of the content categories. Statistically significant differences were observed for costs of PrEP (χ^2^_3_=17.4, *P*=.001) and whether use of PrEP was promoted by the video (χ^2^_3_=13.2, *P*=.004). While videos uploaded by media sources comprised less than 20% of the sample, they were approximately twice as likely to present content related to the cost of PrEP (*P*<.001). Some consumer videos voiced concerns that PrEP would not be affordable for everyone. While the majority of videos reported that the cost is covered by health insurance plans, including Medicare, only a few more recent videos reported the availability of a commercial assistance program that provides free PrEP to people with limited income and no insurance. Compared with videos uploaded by institutions and media, those posted by consumers were less likely to promote the use of PrEP (*P*=.002), whereas those posted by CBOs were more likely to promote the use of PrEP (*P*=.009).

Positive views were emphasized by messages that PrEP is an appropriate prevention strategy for our time. For example, a number of videos oriented toward gay men emphasized that we need to “meet boys where they are.” CBO’s videos, in particular, highlighted a commitment to condom promotion, but acknowledged that many people are not successful in using condoms every time. Hence, these sources emphasized the need for additional prevention strategies. Some videos highlighted that PrEP is a niche opportunity that can be offered safely to people who are at a high risk for HIV infection.

**Table 4:** The most likely target audience of pre-exposure prophylaxis (PrEP) videos (N=217).

Most likely target audience of pre-exposure prophylaxis (PrEP) videos		n (%)	Median # of views	Median # of comments
General population of men who have sex with men (MSM)		83 (38.2)	556^b^	1.7^d^
**Others at risk for HIV categories**^a^		44 (20.3)	706^b,c^	1.3^d,e^
	Racial minority MSM	31 (14.3)	827	1.9
	Transgender population	5 (2.3)	722	1
	Women/heterosexual couples	8 (3.7)	254	0.5
Scientific/professional audience		17 (7.8)	351^c^	0.4^e^
Nonspecific/anyone who may benefit from PrEP/anyone at risk for HIV		73 (33.6)	429^b,c^	0.5^e^
Kruskal-Wallis Test—H (degrees of freedom); *P* value			8.0 (3); .05	19.1 (3); <.001

^a^For Kruskal-Wallis test racial minority MSM, transgender population and Women/heterosexual couples were grouped into one *Others at risk for HIV* category.

^b,c^Superscript letters indicate target audience groups whose mean ranks for views do not differ significantly from each other at the alpha=.05, following post hoc tests.

^d,e^Superscript letters indicate target audience groups whose mean ranks for comments do not differ significantly from each other at the alpha=.05, following post hoc tests.

Less supportive voices expressed the fear of stigmatization such as that those using PrEP will be viewed as “Truvada whores,” or that PrEP is about selfishness and not taking responsibility for practicing safe sex. Additional concerns were raised that individuals with lower levels of income and education will still have hard time accessing the medication or proper health care. Many videos supported a once-a-day pill regimen (ie, “taking one pill a day to stay HIV-negative doesn't seem like asking too much”), and some emphasized that the issue of adherence is secondary (ie, “focusing on adherence is focusing on the wrong side of the problem, as we are meeting people where they are”).

The largest number of videos (98/217, 45.2%) featured a single individual discussing PrEP. These videos were almost universally shot with a single camera, usually with an individual talking directly to the camera. Slightly over a quarter of the videos (60/217, 27.6%) featured multiple individuals, often in the form of interviews, panel discussion or Q&A sessions. Smaller number of videos was either in the form of animation or music videos (25/217, 11.5%) or newsreel and news interviews (20/217, 9.2%). The remainder of the videos (14/217, 6.4%) was in the form of academic presentations accompanied by slides.

Table 4 summarizes the most likely target audience of PrEP videos. To a large extent, videos were directed toward gay and bisexual men (83/217, 38.2%). Racial minority gay and bisexual men were the target of 14.3% (31/217) of the videos. While only 5 videos discussed how PrEP relates to the transgender population specifically, a number of other videos included this population as beneficiaries of PrEP. A PrEP appeal to women was addressed exclusively with 6 videos, and additional 2 videos discussed PrEP in relation to heterosexual serodiscordant couples. The scientific audience was the target of 7.8% (17/217) of reviewed videos. The Kruskal-Wallis test indicated significant differences in both, the number of views (*P*=.05) and the number of comments (*P*<.001) among videos targeting a specific audience. With regard to the number of views, videos appealing to the general population of MSM had a mean rank significantly higher than the videos appealing to a scientific audience (*P*=.04), adjusted for multiple comparisons. With regard to the number of comments posted, videos appealing to the general population of MSM had a mean rank significantly higher than the videos appealing to a scientific audience (*P*=.01), and videos appealing to a general audience (*P*=.002), adjusted for multiple comparisons.

## Discussion

PrEP is the powerful tool for HIV prevention, yet it remains a complex subject with a number of uncertainties concerning implementation. Discussions in literature reinforce the difficulties in communicating reliable and direct information about PrEP to the public [22]. YouTube videos represent a potentially effective approach for communicating reliable information about PrEP to individuals and communities. YouTube can also be a useful instrument for communities to share or retrieve timely health information and advice. However, the health information that is available on YouTube, and social media in general, can also provide contradictory or even misleading information [22,50]. Besides addressing the efficacy and quality of health information content of the YouTube videos [45], researchers can use YouTube videos to investigate public perception of certain diseases, medications, and health care services [54]. To our knowledge, this study represents the first to assess the sources and content of PrEP YouTube videos.

While the first video on PrEP was published in 2009, we observed the surge in the number of published videos in 2014. It is possible that this increase in the number of published PrEP YouTube videos corresponds to pervasive media coverage and to the CDC and WHO clinical practice guidelines issued in 2014. This study indicates that sources of information on PrEP in YouTube videos are diverse. These sources included individuals describing and discussing their experiences while on PrEP, medical professionals providing information about the PrEP regimen, academic institutions offering updates surrounding HIV prophylaxis, CDC providing information on PrEP guidelines, media discussing PrEP as part of a news report, and various CBOs focusing on community dialogue, awareness, and promotion of PrEP.

The most viewed video was published by the CDC, which received the majority of the cumulative total number of views. Interestingly, this video was available online for approximately 9 months only. It is likely that the CDC included this video on their Facebook page and Twitter feed, allowing rapid and expansive reach of the content. While some prior studies of health information in YouTube videos have found that videos uploaded by agencies of the United States Public Health Service are among the most widely viewed [34,44], other studies have shown that videos uploaded by such agencies were not among the most widely viewed [55]. Our results may suggest that viewers are interested in getting reliable and accurate information about PrEP from an authoritative and trusted source. A recent study [56] found that those who learned about PrEP from HIV service agencies and health care professionals were more likely to know a lot about the PrEP medication. Given this finding and the wide reach of YouTube videos, this media channel represents an important way for health care professionals to communicate with the public in ways that will help them make informed decisions about reducing their risk of HIV infection by using PrEP. Considering the ease of access to YouTube, it may be particularly prudent for international organizations and health authorities to consider social media as tools for influencing social and behavioral change in ways that support PrEP uptake and use.

The findings of this study show that YouTube videos cover a wide range of issues associated with PrEP, including intended beneficiaries, how to obtain it, side effects, costs and insurance coverage. The findings highlight that the personal experiences one might have in taking Truvada are covered in YouTube videos. Studies have found that such anecdotal information presented in video format may have an expansive impact on individuals’ health care decisions, extending its benefits from being a diagnostic aid or an educational tool for health care conditions to being a source for information sharing among patients coping with various health issues [57,58]. Hence, it is important for the medical professionals to integrate consumers’ narratives into their messages. The majority of videos have applauded the advent of this new prevention possibility and encouraged those at a risk for HIV to consider this option. However, some videos expressed the views that this intervention is fraught with the unknown. Some voiced concerns about whether Truvada provides a false sense of security, thereby leading to increased rates of sexually transmitted diseases (STDs), and whether users will be exposed to moral judgments, that is, become labeled as “Truvada whores.” Additionally, concerns have been raised regarding the toxicity of the medications and the cost of the treatment. Some, while acknowledging that PrEP is not an intervention for everyone, emphasized that it should be combined with other protective efforts such as using condoms, choosing partners carefully, and monogamy. Yet, by and large, in our sample of most viewed videos, PrEP was promoted and encouraged as a means of HIV prophylaxis, particularly for those at a high risk for HIV infection. 

While we are unable to ascertain the demographics of the viewers, majority of the videos seem oriented toward gay men. Furthermore, these videos had more views and comments than other categories, suggesting that this population has high interest in acquiring information about PrEP. As a group of individuals who face multiple barriers to contact with health professionals, sexual minorities are also more likely than heterosexual people to access the internet at higher rates than heterosexual people to seek health information. For example, one study found that sexual minority participants were 58% more likely to watch a health-related video on YouTube than heterosexual participants [59]. Yet, only a limited number of videos focused on other sexual minority populations at a high risk of contracting HIV, namely the transgender community. A smaller number of videos were directed specifically toward racial minority MSM. None of the videos were directed specifically toward PWID. While previous research has shown that populations that face barriers to contact a health care professional (eg, adolescents, ethnic and racial minorities) are more likely to use the internet to seek health information and to inform their health care decision making [60-63], our findings suggest lack of PrEP information content directed at various other populations at risk. It is, therefore, important to broaden the appeal of PrEP through videos directed at these vulnerable populations.

### Limitations

The findings from this study must be considered in light of the limitations, including the cross-sectional design (popularity based on number of views changes constantly), and the inclusion of videos that had 100 or more views (an arbitrary cut point). By placing this arbitrary cut point we may have created bias toward higher quality and more user-friendly content. While this study represents an important first step in exploring the types of PrEP content available to target audiences, the findings and the insights generated from using predefined categories are somewhat limited. Qualitative exploration of the meaning and scope of these categorizations may provide additional nuances to the issues around PrEP. This data is from a single video-broadcasting website on the internet, as we did not include other video-sharing websites. Although we recorded a number of views for each video in the sample, we have no information on how many unique individuals viewed these videos. YouTube viewers may be more likely to choose videos by default rather than relevance sorting. View counts are an imperfect proxy for measuring the videos’ reach. Furthermore, the study team was also not able to draw conclusions about the possible effects of watching these videos (eg, whether someone decided to seek PrEP upon watching). In addition, the study was based solely on videos in English, the majority of which originated in the United States. The specific comments of viewers were not coded for content. Finally, we did not focus on the additional visual aspects (ie, number of cuts, visual effects, slow motion, bold or unusual colors, and/or intense imagery). Nonetheless, this study contributes to the literature about an emerging topic, namely how social media is providing information related to PrEP use.

### Conclusions

Our study explored PrEP content available on YouTube. The findings demonstrate that content is being uploaded to the site by variety of sources; however, one video from a government source was the most viewed, which may indicate that the public is seeking reliable information about PrEP. Public health professionals should be aware of the extent to which PrEP-related content appears on social media and, more importantly, be attuned to the content, which can be inaccurate or misleading. Future research is needed to identify aspects of YouTube videos that attract viewer attention and best practices for using this medium for increasing public awareness and understanding of PrEP.

## Abbreviations

ARV: antiretroviral
CDC: Centers for Disease Control and Prevention
FDA: Food and Drug Administration
MSM: men who have sex with other men
PrEP: pre-exposure prophylaxis
PWID: people who inject drugs
RA: research assistant
WHO: World Health Organization
